# Quantification of left atrial volume using cardiac magnetic resonance imaging: comparison of left atrial volume index measurements using the Simpson's and bi-plane area-length methods

**DOI:** 10.1186/1532-429X-16-S1-P286

**Published:** 2014-01-16

**Authors:** Hareeprasad R Vongooru, Ashenafi M Tamene, Prabhjot S Nijjar, Sue Duval, Uma Valeti

**Affiliations:** 1 Division of Cardiovascular Diseases, University of Minnesota, Minneapolis, Minnesota, USA

## Background

Increased left atrial volume index (LAVI) has been shown to be an independent predictor of hard cardiovascular events. It can be determined by calculating left atrial (LA) volume through the use of either Simpson's method or area-length method and indexing it to body surface area (BSA). Although there is no standardized method of calculating LA volume using Cardiac Magnetic Resonance Imaging (CMR), the aforementioned methods, which were originally validated for 2-dimensional echocardiography, have been used. We sought to assess the agreement between LAVI, obtained with the use of cardiac gated steady state free precession cine pulse sequence of contiguous short axis slices (LAVI_1_) and area-length method (LAVI_2_).

## Methods

Thirty-six patients (28 males, age 47.6 ± 16.7 years) who had adequate CMR imaging to analyze LA volumes using the two methods were retrospectively identified. LAVI_1 _was calculated using Simpson's stack of discs method on short axis cine images (acquired as 8 mm slices with inter slice gap of 2 mm or 6 mm slices with no gap). LAVI_2 _was measured using the American Society of Echocardiography guidelines. Qmass software (Medis) was used to trace LA areas and volumes.

## Results

**Figure 1:**
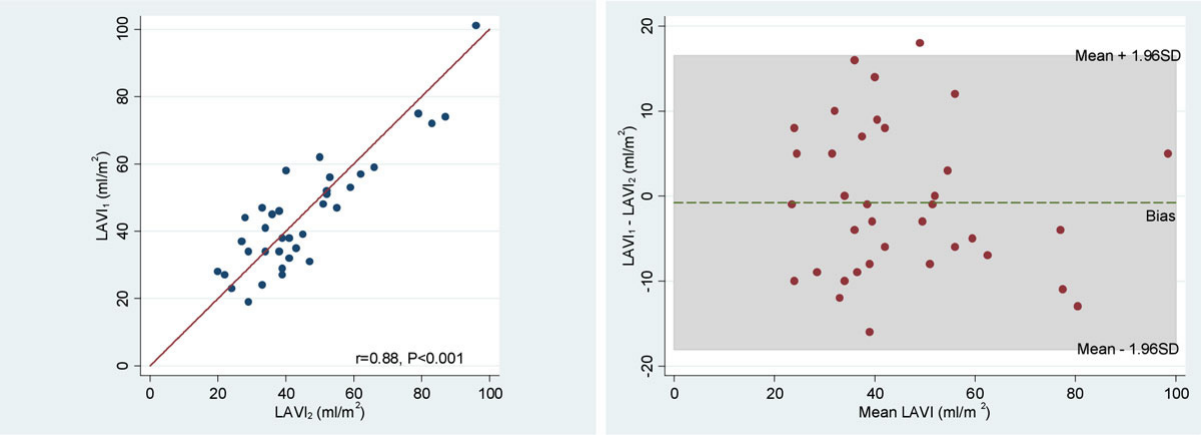
**A: Correlation plot of LAVI1 and LAVI2 Figure 1B: Bland-Altman plot of LAVI1 and LAVI2**.

The mean ( ± SD) values for LAVI_1 _and LAVI_2 _were 44.9 ( ± 17.3) and 45.6 ( ± 18.4) respectively. There was a significant correlation between LAVI_1 _and LAVI_2 _(r = 0.88, p < 0.001) (Figure 1A). Bland Altman analysis showed a mean difference ( ± SD) of -0.75 ± 8.8 mL/m2 between LAVI_1 _and LAVI_2_, with limits of agreement (-18.0, 16.5) (Figure 1B).

## Conclusions

LAVI determined by the Simpson's method of discs and the bi-plane area-length method correlate well in CMR imaging. Our data do not suggest the two methods can be used interchangeably given wide limits of agreement.

## Funding

None.

